# CD56-Positive Acute Myeloid Leukemia Complicated by Hemophagocytic Lymphohistiocytosis: A case report and narrative review

**DOI:** 10.12669/pjms.42.8.16163

**Published:** 2026-08

**Authors:** Xianjie Fu, Ming Li, Chuancai Liu, Yaohui Wu, Junbin Hu, Cong Lu

**Affiliations:** 1 Xianjie Fu Department of Hematology, Central Hospital of Ezhou City, Ezhou, Hubei Province 436000, P.R. China; 2 Ming Li Department of Oncology, Central Hospital of Ezhou City, Ezhou, Hubei Province 436000, P.R. China; 3 Chuancai Liu Department of Hematology, Central Hospital of Ezhou City, Ezhou, Hubei Province 436000, P.R. China; 4 Yaohui Wu, Department of Hematology, Union Hospital, Tongji Medical College of Huazhong, University of Science and Technology, Wuhan, Hubei Province 430022, P.R. China; 5 Junbin Hu, Department of Hematology, Union Hospital, Tongji Medical College of Huazhong, University of Science and Technology, Wuhan, Hubei Province 430022, P.R. China; 6 Cong Lu, Department of Hematology, Union Hospital, Tongji Medical College of Huazhong, University of Science and Technology, Wuhan, Hubei Province 430022, P.R. China

**Keywords:** CD56-Positive, Acute Myeloid Leukemia, Hemophagocytic Lymphohistiocytosis, HLH-94 protocol, BCL-2-targeted therapy

## Abstract

At present, there are no reported cases of CD56+ acute myeloid leukemia (AML) combined with hemophagocytic lymphohistiocytosis (HLH). Meanwhile, there is no standardized treatment plan for AML combined with HLH. This article reports a 27 years old female patient with CD56+ AML who received treatment with an HLH-94-based regimen combined with B cell lymphoma 2 (BCL-2) inhibitor venetoclax. After treatment, the patient’s body temperature returned to normal and laboratory parameters related to HLH improved. Two weeks after chemotherapy, bone marrow reassessment showed 3% blast cells and undetectable MLL-AF9 fusion transcripts; complete remission was subsequently documented on May 23, 2024. To our knowledge, this represents a rarely reported case of CD56+AML with HLH. The result suggests that BCL-2 targeted therapy combined with HLH-94 regimen may be a safe and promising strategy for treating CD56+AML with HLH, and may provide a basis for the selection of treatment strategies for such patients in clinical practice.

## INTRODUCTION

Hemophagocytic lymphohistiocytosis (HLH), also known as hemophagocytic syndrome (HPS), is a life-threatening immune dysregulation disorder.^1^,^2^ The core pathological mechanism of HLH involves abnormal activation and proliferation of macrophages, coupled with functional defects in natural killer (NK) cells and cytotoxic T lymphocytes (CTLs), and manifests as impaired cytotoxic activity, excessive inflammatory responses, and multi-organ damage.^3^ In adults, the most common etiologies of HLH include infections and malignancies, predominantly lymphomas.^4^ Patients with acute myeloid leukemia (AML) may be prone to develop HLH due to impaired immune response due to the disease itself or the associated treatment.^5^ AML-associated HLH is exceedingly rare and occurs in only 10% of patients with AML undergoing intensive chemotherapy.^6^ Therefore, no treatment protocols exist globally for this condition.

CD56 (also known as Neural Cell Adhesion Molecule, NCAM) is a transmembrane glycoprotein that is primarily expressed on the surface of natural killer (NK) cells, some subsets of T lymphocytes, and neural cells.^7^,^8^ Clinical data indicate that positivity is an adverse prognostic factor in AML patients, with its expression significantly associated with chemotherapy response rates, higher relapse risk, and overall survival.^9^,^10^ In this report, we present a case of a patient with CD56+ AML complicated by HLH, effectively treated with B cell lymphoma 2 (BCL-2)-targeted therapy combined with the HLH-94 protocol. Published reports specifically describing CD56-positive AML complicated by clinically diagnosed HLH appear to be extremely limited. In conjunction with a narrative review on this rare disease, this study aimed to enhance clinical awareness of the disease and provide insights for the selection of treatment strategies.

## CASE PRESENTATION

A 27 years old woman visited the fever clinic with the major complaint of “fever and nausea” on March 30, 2024. Outpatient blood routine examination showed: white blood cell count of 33.66 * 109/L, lymphocyte count of 3.84 * 109/L, monocyte count of 23.81 * 109/L, red blood cell count of 4.23 * 1012/L, hemoglobin 120.00g/L, platelet count 70.00 * 109/L, with notes indicating the presence of cells of unknown classification. After empirical treatment with levofloxacin lactate and peramivir in the outpatient department, the patient continued to experience intermittent high fever (peak temperature: 39.5°C) and no other symptoms, such as cough or sputum production. On March 31, 2024, the patient was diagnosed with “mononucleosis with thrombocytopenia and fever of unknown origin (FUO)” and was transferred to the hematology department for specialized evaluation and management.

### Physical examination upon admission:

body temperature of 38.2, no yellow staining on the skin and sclera, no new bruising or spots observed. Several lymph nodes of varying sizes can be palpated in the armpits and groin, with a maximum diameter of approximately 2cm, palpable 2cm below the splenic rib margin.

### Blood biochemical examination:

**Fig.1:**
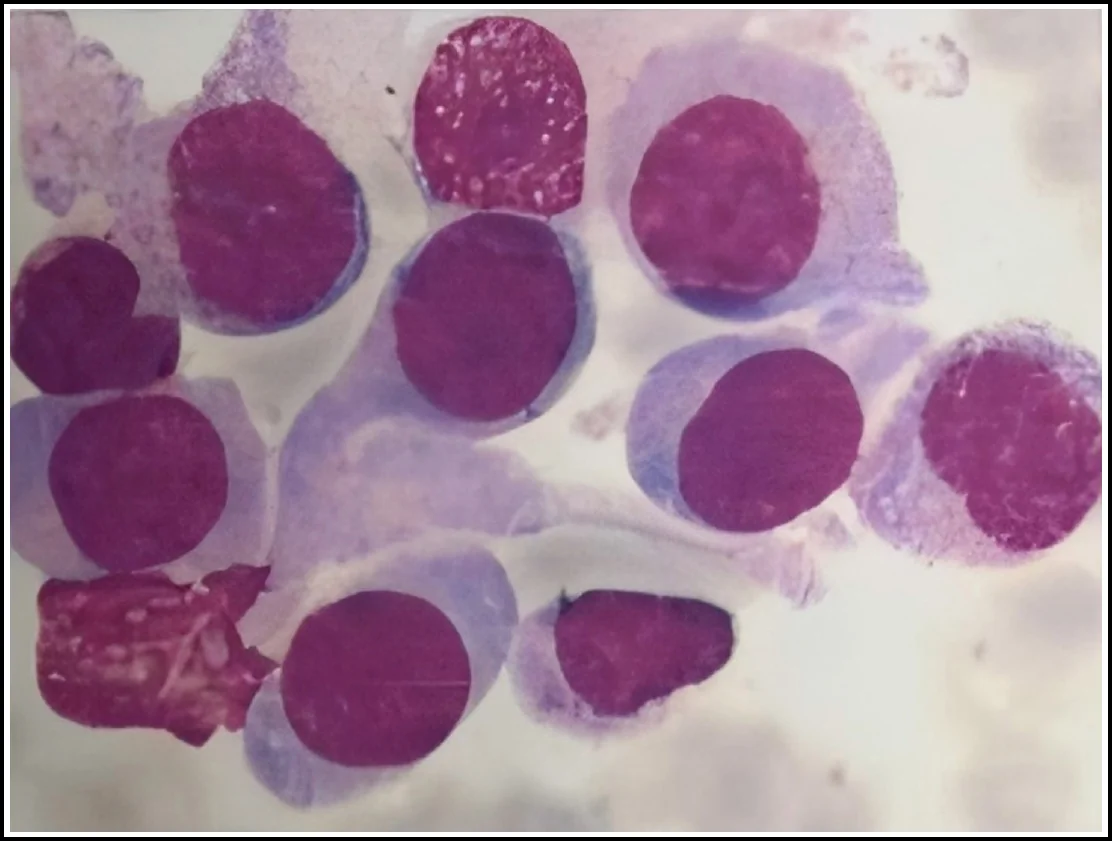
Bone marrow cell morphology in patients with AML complicated with HLH, visualized by bone marrow smear staining. A large number of abnormal blast cells, which have large nuclei, fine nuclear chromatin, prominent nucleoli, and scant cytoplasm, indicative of a typical AML cell morphology.

Alanine aminotransferase 174.00U/L, Aspartate aminotransferase 135.00U/L, Lactate dehydrogenase 1546.00U/L, Human chorionic gonadotropin<2.00IU/L. Coagulation test showed: prothrombin time (PT) 13.8 s (PT control 13.5 s), fibrinogen (FIB) 1.90 g/L, D-dimer 30.58 mg/L. High sensitivity C-reactive protein 8.4 mg/L, procalcitonin 1.122ng/ml. HLH-related tests showed that ferritin was>1650ng/mL and NK cell activity was normal. Novel coronavirus nucleic acid RNA detection, respiratory virus nucleic acid detection (13 items), blood culture, plasma cytomegalovirus (CMV), and Epstein-Barr Virus (EBV) tests were all negative. Imaging examination: chest computer tomography (CT) detected small nodules in the lower lobe of the patient’s left lung and possible small lymph nodes in the lungs. Bone marrow cytology showed markedly hypercellular marrow with findings suggestive of acute leukemia, while AML required further confirmation (Fig.1). Bone marrow nucleated cell proliferation was highly active, with unclassified cells accounting for 85% of bone marrow nucleated cells and 77% of peripheral blood cells on Wright–Giemsa-stained smears.. Bone marrow biopsy showed extremely active bone marrow proliferation (90%), high granulocyte to erythroid ratio, significant proliferation of the granulocyte system, mainly immature granulocytes, and decreased erythroid and megakaryocytes. Combined with immunohistochemistry, the diagnosis of AML was made.

**Fig.2:**
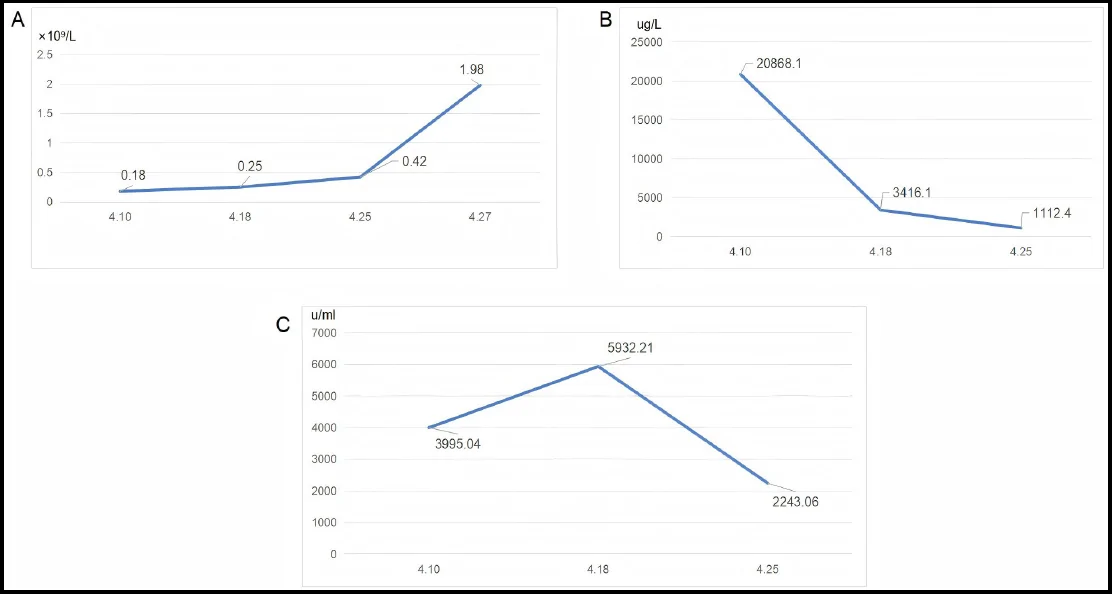
A. Trend chart of white blood cell count changes; B. Trend chart of ferritin concentration changes; C. Trend chart of soluble interleukin-2 receptor (sCD25) concentration changes.

Flow cytometry examination showed that abnormal cells accounted for approximately 87.5% of all nucleated cells, with a myeloid phenotype mainly expressing CD13, HLA-DR, CD56 (81%), CD64, CD33, CD4, and CD38, suggesting possible AML with atypical phenotype (Fig.2). Chromosome karyotype was as follows: 47, XX, +8[18]/46, XX[2].

### Leukemia gene screening:

After screening 46 common fusion genes in leukemia, it was found that the MLL-AF9 fusion gene was positive, which is consistent with the clinical significance and incidence of the MLL-AF9 fusion gene in AML.^11^ The patient was transferred to the Union Hospital, Tongji Medical College of Huazhong, University of Science and Technology on April 9, 2024. A review of the bone marrow morphology showed hyperplasia of nucleated cells, with monocytes accounting for 91.5%, of which 84% were primitive monocytes. Erythroblasts were rare, megakaryocytes were not seen, and a large number of leukemia cells were seen in the peripheral blood.

Considering the patient’s persistent fever (>38.5°C for ≥7 days), progressive decline in complete blood counts (hemoglobin <90 g/L, platelets <100×10/L, neutrophils <1.0×10/L) and fibrinogen (<1.5 g/L), progressive elevation of transaminases and ferritin (≥500μg/L), significantly elevated sCD25 (>2400 U/mL), abnormal coagulation function, and splenomegaly, secondary HLH was diagnosed. Treatment was initiated on April 12 as follows: venetoclax 100mg daily on days 1-21 (co-administered with posaconazole); etoposide regimen (etoposide 100mg/m² on day one weekly); Dexamethasone: 10 mg/m^2^ in weeks 1-2, 5 mg/m^2^ in weeks 3-4, 2.5 mg/m^2^ in weeks 5-6, and 1.25 mg/m^2^ in weeks 7-8. The patient’s body temperature gradually returned to normal. Bone marrow examination was repeated two weeks post-chemotherapy and showed 3% blast cells and the negative MLL-AF9 fusion gene status. After treatment, white blood cells showed an upward trend until granulocyte deficiency was compensated; sCD25 and ferritin significantly decreased (Fig.2). This suggests timely control of hemophagocytic syndrome after chemotherapy. On May 23, 2024, treatment response assessment by bone marrow aspiration indicated complete remission (CR).

### Ethical approval:

The ethics committee of our hospital approved this case report (No. 2026-0081, Date: February 2^nd^ 2026), and written informed consent has been obtained from the involved patient.

## NARRATIVE REVIEW

### Diagnostic Criteria for HLH and Treatment Strategies for AML:

The diagnosis of HLH relies on two main aspects: genetic testing identifying pathogenic mutations in relevant genes (e.g., PRF1, UNC13D), which provides a definitive diagnosis. Fulfilling ≥5 of the clinical/laboratory criteria from the HLH-2004 protocol,^12^ including: persistent fever (>38.5°C for ≥7 days), splenomegaly, bicytopenia or pancytopenia, hypertriglyceridemia (fasting triglycerides ≥3.0 mmol/L) and/or hypofibrinogenemia (fibrinogen ≤1.5 g/L), serum ferritin >500 μg/L, plasma soluble CD25 >2400 U/mL, reduced/absent NK-cell activity, hemophagocytosis identified in bone marrow, spleen, or lymph nodes. Among these criteria, CD25 (sCD25) levels and cell activity assays exhibit high specificity.^13^ Notably, ferritin >10,000μg/L achieves >90% diagnostic sensitivity and specificity, providing critical guidance for early identification of HLH.^14^

Venetoclax (as monotherapy or combined with hypomethylating agents like azacitidine) demonstrates significant efficacy in treating AML. It acts by inhibiting the anti-apoptotic protein BCL-2, thereby sensitizing tumor cells to apoptotic signals, and has a favorable tolerability.^15^ This approach offers a novel option for patients who are resistant to conventional chemotherapy or unfit for intensive regimens, carrying significant clinical importance for elderly populations.

### Expression of CD56 in AML and Related Research:

CD56 expression is detectable in approximately 15%-20% of AML patients.^16^ However, the CD56 positivity rate is significantly higher in AML patients with the t(8;21) chromosomal translocation, reaching up to 65%.^17^–^19^ Current research indicates that CD56+ leukemic blasts are closely associated with extramedullary infiltration (EMI) and lymphadenopathy in AML.^20^ Flow cytometry analysis demonstrated that CD56+ patients are more prone to EMI (e.g., granulocytic sarcoma) and often exhibit 11q23 karyotype abnormalities, lower complete remission rates, and shorter overall survival, suggesting CD56’s utility as a significant prognostic marker in AML.^20^ Furthermore, CD56+ cells may originate from a less differentiated leukemic stem cell population.^21^ CD56 expression and increased expression of multidrug resistance genes, such as MDR1, have been associated with chemoresistance, relapse risk, and poor prognosis in AML.^22^,^23^ Overexpression of CD56 is significantly correlated with drug resistance in AML treatment and disease progression. Studies by Sasca D et al. and Xu S et al.^16^,^24^ indicate that CD56+ leukemia cells exhibit reduced sensitivity to chemotherapeutic drugs and kinase inhibitors, likely due to enhanced drug efflux mediated by MDR1. Additionally, CD56 has been associated with tumor cell adhesion and migration, increasing the risk of central nervous system and skin infiltration in both lymphoma and AML.^25^ Clinical data demonstrate that CD56 positivity is an independent adverse prognostic factor in AML patients, with its expression significantly associated with low chemotherapy response rates, high relapse risk, and shortened overall survival.^9^,^17^,^26^,^27^ Therefore, the expression status of CD56 can serve as a key indicator for evaluating AML patient prognosis, aiding clinicians in making more precise judgments about patient conditions during treatment decision-making.

### Immunological Mechanism of HLH:

The core pathological mechanism of HLH is immune dysregulation caused by a defective function of cytotoxic lymphocytes (CTL) and NK cells.^28^ Under normal conditions, CTL/NK cells eliminate abnormal cells by releasing perforin and granzymes.^29^ In HLH, however, mutations in genes such as PRF1 and UNC13D can impair cytotoxic function, leading to persistent antigen presence, a cytokine storm, macrophage activation, and phagocytosis of blood cells.^14^ Recent studies have also found that mutations in genes like NLRC4 can induce similar pathological processes, indicating high heterogeneity in the immune mechanisms underlying HLH.^30^

### Research Status of AML Complicated by HLH:

AML complicated by HLH is clinically rare and carries an extremely poor prognosis,^31^ and unclear underlying mechanisms. Secondary HLH is often associated with infections, malignancies, or autoimmune diseases, while AML complicated by HLH may involve leukemia cell-induced dysregulation of the immune microenvironment.^32^ CD56+ AML cells might be associated with the development of HLH through adhesion molecule-mediated immune escape and the secretion of inflammatory factors.^20^ Clinical management requires addressing both AML chemotherapy and controlling HLH-associated inflammation. BCL-2 inhibitors combined with chemotherapy or hypomethylating agents show synergistic potential, while hematopoietic stem cell transplantation (HSCT) may offer a curative opportunity for high-risk patients.^15^

This narrative review summarizes available research on the expression of CD56 in AML, as well as on HLH. It provides clinicians with important reference information on these two diseases, thereby improving their understanding and comprehension of the conditions and allowing more substantial support for patient diagnosis and treatment.

## DISCUSSION

This study reports a case of a CD56+ AML patient with HLH who underwent treatment with the HLH-94 protocol combined with the BCL-2 inhibitor venetoclax. CD56, as a neural cell adhesion molecule (NCAM), may contribute to the pathological process of AML complicated by HLH through multiple mechanisms. First, CD56 may contribute to the interaction between tumor cells and the microenvironment, inhibiting the killing function of CTLs/NK cells and facilitating immune escape.^33^ Second, CD56+ cells may promote pro-inflammatory cytokines such as IL-1β and IL-6, exacerbating the cytokine storm.^34^ Additionally,CD56 has been associated with the infiltration of leukemia cells into organs like the liver and spleen, directly inducing tissue damage and inflammatory responses.^17^,^20^ These mechanisms collectively form the complex pathological network of CD56+ AML complicated by HLH, although its specific regulatory pathways still require in-depth exploration. The proposed mechanisms linking CD56 expression to HLH are based on previously published studies and remain speculative in the context of a single case report.

High expression of CD56 may be associated with the pathological process of AML complicated by HLH through the following molecular mechanisms:

Immune escape and inflammatory activation. As NCAM, CD56 may mediate the adhesion of AML cells to the microenvironment, inhibiting the cytotoxic function of NK cells and cytotoxic T lymphocytes (CTLs), thereby leading to failure of immune surveillance.^35^ Simultaneously, CD56+ AML cells may directly exacerbate the cytokine storm and promote the occurrence of HLH by secreting pro-inflammatory cytokines such as IL-6 and TNF-α.^36^

Drug resistance and disease progression.^37^ CD56 overexpression is associated with the upregulation of multidrug resistance genes (e.g., MDR1). It may enhance drug efflux or activate survival signaling pathways such as PI3K/AKT, leading to chemoresistance in AML cells.^37^ This, in turn, aggravates myelosuppression and immune dysregulation, further worsening the prognosis of HLH.

Patients with AML complicated by HLH often present with nonspecific symptoms, such as fever and cytopenia, which are easily misdiagnosed as infection or chemotherapy side effects.^6^ In this case, although infectious causes were extensively investigated, the patient met five HLH-2004 diagnostic criteria and was ultimately diagnosed with HLH. The diagnosis of HLH was based on fulfillment of HLH-2004 criteria, while flow cytometry confirmed the presence of CD56-positive AML.^14^ This study suggests that clinicians should maintain vigilance for HLH complicating CD56+ AML. This necessitates integrating multimodal diagnostic approaches, including cytometry, genetic sequencing, and bone marrow pathology, and collaboration among specialists in hematology, radiology, and immunology.^20^

Treatment of CD56+ AML complicated by HLH requires simultaneous suppression of leukemia proliferation and the excessive immune response. In this patient, venetoclax was administered together with an HLH-directed regimen, and this combination was associated with control of hyperinflammation and subsequent hematologic remission. It induces apoptosis by targeting BCL-2 while modulating the immune microenvironment. For cytokine storm, immunomodulatory drugs such as etoposide and dexamethasone can be added. Patients with poor response to induction therapy should undergo early evaluation for allogeneic hematopoietic stem cell transplantation.^15^ Furthermore, dynamic monitoring of CD56 expression levels aids in assessing treatment response and prognosis.^17^,^26^

This case report showed that an HLH-94-based regimen combined with the BCL-2 inhibitor venetoclax demonstrated a synergistic effect. Etoposide induces apoptosis in tumor cells by inhibiting DNA topoisomerase II, while simultaneously suppressing macrophage overactivation.^38^ Dexamethasone can rapidly controls cytokine release. Venetoclax overcomes the apoptotic resistance of AML cells by targeting the BCL-2 protein.^39^ Therefore, these agents may provide complementary therapeutic effects by targeting AML-cell survival pathways while simultaneously suppressing HLH-associated hyperinflammation. Previous evidence also suggests that venetoclax-based therapy may have potential clinical value in AML-associated HLH; however, its efficacy requires confirmation in larger studies.^40^ This regimen may improve response rates in CD56+ AML patients with HLH.

To our knowledge, this is the first case of CD56+AML with HLH. The findings of this case report may serve as a basis for the selection of treatment strategies for such patients in clinical practice and provide evidence for future research. However, the long-term efficacy and risk of resistance of the treatment regimen was not validated in this case report. Future research needs to further clarify the specific role of CD56 in HLH development and its regulatory relationship with MDR1, and explore the potential application of CD56 monoclonal antibodies or Chimeric Antigen Receptor (CAR)-T cell therapy in treatment-resistant patients. Multicenter clinical studies are required to validate the long-term efficacy and safety of BCL-2 inhibitor combination regimens with immunomodulatory agents.

## CONCLUSIONS

CD56 expression holds significant importance in the diagnosis and treatment of AML complicated by HLH. High CD56 expression, leading to dysregulated immune responses and resistance mechanisms, may represent a potential biomarker for AML with HLH. BCL-2-targeted combination therapies may offer novel therapeutic avenues for this patient population. A deeper understanding of the mechanistic role of CD56 in AML may provide clues for developing new treatment strategies. Future research should further investigate specific functions of CD56 in AML pathogenesis and its potential applications in managing AML with HLH, aiming to deliver more precision-based and effective therapeutic strategies for this patient population.

### Authors’ contributions:

**XF and ML:** Literature search, study design and manuscript writing. **CL, YW** and **JH:** Data collection, data analysis and interpretation. Critical Review. **XF** and **ML:** Manuscript revision and validation and is responsible for the integrity of the study. All authors have read and approved the final manuscript.
